# New Insight into Immunity and Immunopathology of Rickettsial Diseases

**DOI:** 10.1155/2012/967852

**Published:** 2011-09-06

**Authors:** Pasquale Mansueto, Giustina Vitale, Antonio Cascio, Aurelio Seidita, Ilenia Pepe, Antonio Carroccio, Salvatore di Rosa, Giovam Battista Rini, Enrico Cillari, David H. Walker

**Affiliations:** ^1^Dipartimento di Medicina Clinica e delle Patologie Emergenti, Universitá di Palermo, Via del Vespro 141, 90127 Palermo, Italy; ^2^Dipartimento di Patologia Umana, Universitá di Messina, Via Consolare Valeria 1, 98125 Messina, Italy; ^3^Unità Operativa Complessa di Medicina Interna, Ospedali Civili Riuniti di Sciacca and Università di Palermo, Via Pompei, 92019 Agrigento, Italy; ^4^Azienda Ospedaliera “Villa Sofia-Cervello”, Viale Strasburgo 233, 90146 Palermo, Italy; ^5^Unità Operativa Complessa di Patologia Clinica, Azienda Ospedaliera “Villa Sofia-Cervello”, P.O. “V. Cervello”, Via Trabucco 180, 90146 Palermo, Italy; ^6^The Carmage and Martha Walls Distinguished University Chair in Tropical Diseases, University of Texas Medical Branch, University Boulevard 301, TX 77555 Galveston, USA

## Abstract

Human rickettsial diseases comprise a variety of clinical entities caused by microorganisms belonging to the genera *Rickettsia*, *Orientia*, *Ehrlichia*, and *Anaplasma*. These microorganisms are characterized by a strictly intracellular location which has, for long, impaired their detailed study. In this paper, the critical steps taken by these microorganisms to play their pathogenic roles are discussed in detail on the basis of recent advances in our understanding of molecular *Rickettsia*-host interactions, preferential target cells, virulence mechanisms, three-dimensional structures of bacteria effector proteins, upstream signalling pathways and signal transduction systems, and modulation of gene expression. The roles of innate and adaptive immune responses are discussed, and potential new targets for therapies to block host-pathogen interactions and pathogen virulence mechanisms are considered.

## 1. Introduction

The human rickettsial diseases are a variety of clinical entities caused by *α*-proteobacteria of the order *Rickettsiales* belonging to genera *Rickettsia *and *Orientia* (family: *Rickettsiaceae*) and *Ehrlichia* and *Anaplasma *(family: *Anaplasmataceae*) [1, 2]. 

Rickettsiae are small, obligately intracellular, gram-negative bacteria that reside free in the host cell cytoplasm, and are transmitted to human hosts by arthropod vectors. A modern classification based on whole-genome analysis, divides the species of the genus *Rickettsia *in four groups: spotted fever group (*R. rickettsii*, *R. conorii*, *R. parkeri*, and several others), typhus group (*R. prowazekii *and* R. typhi*), ancestral group (*R. bellii *and *R. canadensis*, not known to be pathogenic), and transitional group (*R. akari*, *R. australis*, and *R. felis*). Further investigation of a variety of hosts including nonhematophagous insects, amoebae, and leeches identified many novel *Rickettsia *clades opening a broader view of their evolution [3].

*Rickettsia prowazekii *is the only pathogen among various rickettsial species with acknowledged capacity to maintain persistent subclinical infection in convalescent patients, which can later manifest as recrudescent typhus or Brill-Zinsser disease. 

*Orientia tsutsugamushi* is the etiologic agent of scrub typhus, a rickettsiosis that is widespread in Asia, the islands of the western Pacific and Indian Oceans, and foci in northern Australia. The genus *Ehrlichia* is member of the family *Anaplasmataceae*, which also includes the genera *Anaplasma*, *Wolbachia,* and *Neorickettsia*. It consists of six formally named members: *E. canis*, *E. chaffeensis*, *E. ewingii*,* E. muris*, *E. Ovis,* and *E. ruminantium*. Human monocytic ehrlichiosis, caused by *E. chaffeensis*, and human granulocytic anaplasmosis, caused by *Anaplasma phagocytophilum*, are the most important ehrlichioses and are considered emerging diseases. Major findings in rickettsioses and ehrlichioses/anaplasmosis include fever in a patient with exposure to a potential vector that may be associated with rash, inoculation eschar, or localized lymphadenopathy. Laboratory studies commonly reveal neutropenia, thrombocytopenia, and moderate increases in hepatic transaminases. The severity of rickettsial diseases varies with the causative agent and the host. Older age, alcoholism, and deficit in glucose-6-phosphate dehydrogenase have been associated with more severe disease [1, 2].

For each genus, the main steps taken by the microorganism to play their pathogenic roles will be discussed with particular emphasis to the roles of innate and adaptive immune responses.

## 2. Rickettsia spp

### 2.1. Endothelial Cell Invasion, Injury, and Activation

Although pathogenic rickettsiae are able to infect and replicate *in vitro* in a great number of different cell types, during *in vivo* infection, both in humans and in established experimental models of infection, the pathogens invade and proliferate within vascular endothelial cells (ECs) lining small and medium-sized blood vessels, the major target cells of rickettsial infection, together, to a lesser extent, with perivascular cells, that is, monocytes and macrophages, and hepatocytes, destroying them, spreading infection to the endothelia of the vascular tree. The recently emerging concept of “ECs heterogeneity” considers the significant morphological and functional differences between ECs of small and large vessels and between cells derived from various microvascular endothelial beds, and the necessity for a more comprehensive analysis of the biological basis of rickettsial affinity for vascular ECs (see below). However, damage to the endothelium, and subsequent endothelial dysfunction and activation, is followed by acute phase responses and alteration in coagulation and in the cytokine network, together with transient immune dysregulation, characterized by reduction in circulating peripheral CD4+ T lymphocytes and perivascular infiltration by CD4 and CD8 T lymphocytes, B cells, and macrophages, all features collectively termed as “rickettsial vasculitis”. The mechanisms of host defence are not yet completely understood, although cell-mediated immunity is thought to play a crucial role [4, 5]. 

#### 2.1.1. ECs Invasion

**Figure 1:**
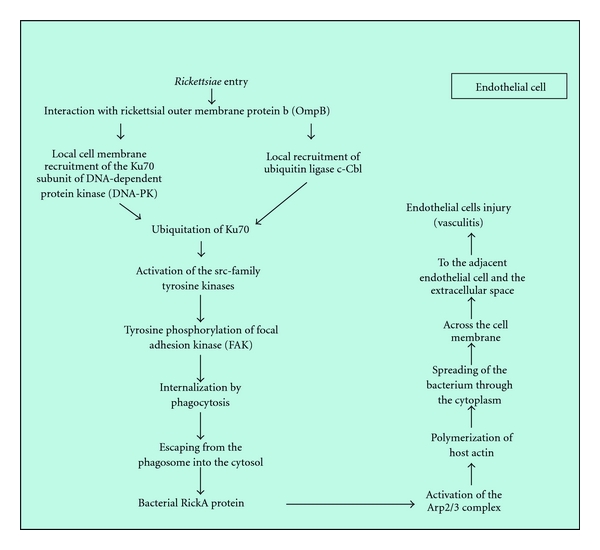
Rickettsia entry into endothelial cells.

Rickettsial entry into human ECs via induced phagocytosis requires rickettsial viability and effector function of the host cell cytoskeletal actin, implicating that adherence of a viable bacterium to the cell surface triggers intracellular uptake by a metabolically active host cell. Even though some signalling events involved in rickettsial entry into the ECs have been documented, that is, activation of phosphoinositide 3-kinase, Cdc42 (a small GTPase), src-family tyrosine kinases, and tyrosine phosphorylation of focal adhesion kinase (FAK) and cortactin, all of the effector host proteins mediating entry are not fully elucidated (Figure 1) [6]. 

Some studies, especially on spotted fever group rickettsiae* (R. rickettsii *and* R. conorii)*, have reported the Ku70 subunit of a mainly nuclear DNA-dependent protein kinase, located in cytoplasm and plasma membrane too, as a receptor involved in rickettsial internalization. Ku70 is recruited to rickettsial entry sites, and inhibition of its expression impairs rickettsial internalization. Rickettsial infection also stimulates the ubiquitination of this kinase. The ubiquitin ligase c-Cbl is recruited to rickettsial entry foci, and downregulation of c-Cbl blocks rickettsial invasion and Ku70 ubiquitination. Experimental approaches identified the rickettsial outer membrane protein B (OmpB) as a ligand for Ku70 and that Ku70-OmpB interactions are sufficient to mediate rickettsial invasion of nonphagocytic host cells. Furthermore spotted fever group rickettsiae use OmpA, Sca1, and Sca2 as adhesion proteins [7]. Antibodies to particular epitopes of OmpA and OmpB may protect against reinfection, but they appear not to play a key role in immunity against primary infection [8]. OmpA, OmpB, Sca1, and Sca2 belong to a family of outer membrane proteins, termed autotransporters, found in gram-negative bacteria. Bioinformatic analysis of sequenced rickettsial genomes has identified a family of at least 15 genes in addition to *ompA* (*sca0*) and *ompB* (*sca5*), termed *sca *(for surface cell antigens), whose predicted products resemble autotransporter proteins. The function of all of these Sca proteins remains unknown. However, it was hypothesized that they should contribute to the specific recognition of different sets of host receptors. It is possible that Sca1 and Sca2 may work in concert with other rickettsial proteins (i.e., OmpB and OmpA) to interact with target mammalian cells, especially ECs during the infection process. The interactions of Sca1 and Sca2 with their cognate receptors may be coordinated with other receptor-ligand pairs to trigger previously observed downstream signaling events, that is, phosphoinositide 3-kinase, Cdc42, and src-family tyrosine kinases that, ultimately, lead to localized actin recruitment and nucleation required for entry [9–11].

Other possible rickettsial adhesins, encoded by the genes RC1281 and RC1282 in *R. conorii*, and, respectively, designed as Adr1 and Adr2, have been proposed to be also involved in bacterial adhesion and entry into the host cells [12].

The rickettsiae induce internalization by phagocytosis. Then, to enter the cytosol of the host cell, where nutrients, adenosine triphosphate, amino acids, and nucleotides, are available for growth, and to avoid phagolysosomal fusion and death, rickettsiae must escape from the phagosome. Rickettsiae secrete phospholipase D and heamolysin C, encoded by the rickettsial genes *pld* and *thyC *which are capable of disrupting the phagosomal membrane and allowing a rapid escape. However, knockout of the *pld* gene alone does not prevent phagosomal escape [13].

In the cytosol, rickettsiae express a surface protein, Sca2, which recruits the Arp2/3 complex, activate it inducing directional host actin polymerization, which, finally, propels the bacterium through the cytoplasm and across the cell membrane into the adjacent endothelial cell or extracellular space. On its own, the Arp2/3 complex has no actin nucleation activity. It is activated by a class of cellular proteins called nucleation-promoting factors (NPFs), two examples of which are members of the Wiskott-Aldrich syndrome protein (WASP) family and of the cortactin family. RickA, a mimic of WASP family NPFs, has been proposed as the mediator of Arp2/3 recruitment and actin nucleation. However, the evidence for a role for Sca2 including gene knockouts and complementation is more extensive and compelling [14].

Then filaments of actin propel *Rickettsia *to the surface of the host cell, where host cell membrane is deformed outward and invaginates into the adjacent cell. Disruption of both cell membranes enables the *Rickettsia *to enter the adjoining cells without being exposed to the extracellular environment. However, some rickettsiae exit via the luminal surface of blood vessels into the bloodstream [15, 16].

Typhus group rickettsiae tend to accumulate within the cytoplasm until cell lysis as *R. prowazekii* does not stimulate actin-based mobility and *R. typhi *exhibits only erratic mobility. Spotted fever group organisms spread rapidly from cell to cell and usually accumulate intracellularly in significantly lower numbers than typhus group rickettsiae. It would be reasonable to hypothesize that the host cell is able to sustain growth, multiplication, and accumulation of progeny rickettsiae during infection with typhus group organisms, owing to significant differences in pathogenic mechanisms of cell injury in comparison to those triggered by spotted fever group rickettsiae, that is, differences in the intensity and/or kinetics of the nuclear-factor- (NF-) *κ*B activation pattern in *R. Typhi- *versus* R. conorii*-infected host ECs (see below) or stimulation of oxidative stress [17]. 

In addition to the above-mentioned rickettsial mechanisms of EC invasion, several studies pointed out the role of T4SS as an additional virulence factor. T4SSs are complex multiprotein structures spanning the bacterial envelope and are composed of up to 12 individual protein components classified into three groups, each represented in this bacterial genus. The exact function of these transporters, which are ancestrally related to bacterial conjugation systems, diverged during evolution. Since T4SS components are retained in rickettsial genomes, many of which lack plasmids; their primary suspected role is secretion of virulence factors rather than conjugation. Among rickettsial substrates susceptible to be exported by such a secretion system are sec7 proteins (effectors known to contribute to the establishment of a replicative organelle by inhibiting phagosome-lysosome fusion, even if these bacteria grow in the cytoplasm of infected cells), LepA (a protein promoting release of bacteria from protozoa by an exocytic pathway, a marker of probable ancestral location of rickettsiae within ameba), and, finally, VipD (a protein belonging to the patatin family of proteins, with phospholipase A_2 _[PLA2] activity that perturbs membrane trafficking and modulates intracellular bacterial growth) [18]. 

Factors secreted by the T4SS most likely promote rickettsial survival by triggering synthesis of nutrients from the host cell or allowing adaptation of rickettsiae to the intracellular environment [19].

#### 2.1.2. ECs Injury

**Figure 2:**
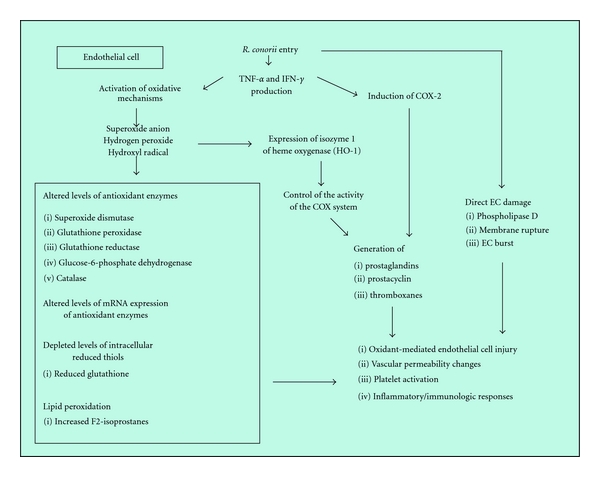
Rickettsia injury to endothelial cells.

The mechanisms responsible for cell injury and vascular denudation by rickettsiae remain poorly understood (Figure 2) [20]. A comprehensive analysis of damage to the membrane structure and integrity of subcellular organelles by electron microscopy indicated the possibility of involvement of reactive oxygen species (ROS) production, that is, superoxide anion (O_2_^−^), hydrogen peroxide (H_2_O_2_), and hydroxyl radical (OH^−^), induced by the combination of tumor-necrosis-factor- (TNF-) *α* and interferon- (IFN-) *γ*, in rickettsial pathogenesis, both as host defense and infection-induced injury mechanisms. *In vitro* and *in vivo* experiments demonstrated the induction of oxidative stress mechanisms, as evidenced by accumulation of ROS, altered levels of antioxidant enzymes, that is, superoxide dismutase, glutathione peroxidase, glutathione reductase, glucose-6-phosphate dehydrogenase (G6PD), and catalase, alterations in mRNA expression of selected antioxidant enzymes, and depleted levels of intracellular reduced thiols, for example, reduced glutathione (*γ*-glutamylcysteinylglycine). These studies suggest an important role for oxidant-mediated cell injury in the pathogenesis of rickettsial infections. In addition, compared to subjects expressing physiologic levels of G6PD, the prognosis in G6PD- deficient patients is relatively poor, due to complications, such as hemolysis (involving red blood cells which lack the ability to synthesize G6PD to replace unstable prematurely denatured G6PD) and acute renal failure (as consequence of hemolysis), and enhanced severity of disease. These observations confirm the potential importance of antioxidant defence in the outcome of human rickettsial infections [21, 22]. In this context, it has also been demonstrated that rickettsial infection *in vitro* as well as *in vivo* induces the expression of hemeoxygenase-1 (HO-1), the inducible form of antioxidant and vasoprotective isozyme 1 of heme oxygenase, a rate-limiting enzyme in the pathway for heme catabolism, resulting in the release of ferrous iron and generation of carbon monoxide (CO) and biliverdin. The latter is subsequently converted to bilirubin. One of the most critical regulatory functions of HO-1 in the vasculature is to control the activity of the cyclooxygenase (COX) system, which is responsible for generation of a number of vasoactive substances, including prostaglandins (PGs), prostacyclin, and thromboxanes. Rickettsial infection of human ECs causes robust induction of COX-2 mRNA and protein expression but has no apparent effect on the constitutive COX-1 isoform. Induction of the endothelial COX-2 system and the resultant enhanced release of vasoactive PGs, especially PGE2 and PGI2, may contribute to the regulation of inflammatory responses and vascular permeability changes during rickettsial infection [23, 24]. 

Cell injury and vascular denudation of the endothelium may also result from direct damage to host cells. One among possible mechanisms of direct damage is a rickettsial phospholipase activity, that is, phospholipase D or phospholipase A_2_, that may contribute to direct host damage. Spotted fever group rickettsiae may also damage cells at the time of exiting, when directional actin polymerization is followed by membrane rupture. Otherwise, typhus group rickettsiae, which lack directional actin polymerization (*R. typhi *polymerizes actin, but not directionally), grow inside infected cells until the cells burst, and, in this context, genes, encoding proteins with possible membranolytic activities, for example, pat1 and tlyC, could play a role [25]. Another widely accepted concept regarding the pathogenesis of EC injury and rickettsial diseases is increased vascular permeability, resulting in fluid imbalance and edema of vital organs, as a major feature of acute inflammation.* R. conorii *infection of human ECs induces changes in the localization and staining patterns of adherens junction proteins, and discontinuous adherens junctions lead to formation of interendothelial gaps. Interestingly, proinflammatory cytokines, that is, TNF-*α* and IFN-*γ*, during infection, exacerbate the effects of rickettsiae on endothelial permeability, further suggesting that the changes in the barrier properties of vascular endothelium are likely due to a combinatorial effect of intracellular rickettsiae and immune responses mediated by and/or directed against the infected host cell [26]. 

#### 2.1.3. EC Activation: Intracellular Signaling Mechanisms

It is widely accepted that interactions between invading rickettsiae and host ECs constitute one of the most important aspects underlying the onset and progression of infection, replication within the intracytoplasmic niche, dissemination through the host, and the pathogenesis of resultant disease. *In vitro* studies suggest that ECs undergo a series of active cellular responses during infection with rickettsiae, resulting in significant alterations in the pattern of gene transcription and characteristic responses, a phenomenon referred to as “endothelial activation” [20]. 

Many pathophysiological situations affecting ECs of the vasculature, for example, rickettsial invasion, lead to the expression of genes dependent on the NF-*κ*B family of transcription factors. Most of the early-response genes transcriptionally upregulated in response to rickettsial invasion, that is, IL-8 and Monocyte-Chemoattractant-Protein-(MCP-) 1 (see below), contain NF-*κ*B binding sites in their promoter regions, indicating that infection-induced alterations in gene expression may be governed, at least in part, by activation of NF-*κ*B. Human ECs infected with rickettsiae display nuclear translocation of NF-*κ*B. This event is a consequence of the degradation, by proteasomes, of masking regulatory proteins, termed inhibitors of NF-*κ*B, or I*κ*B, after their phosphorylation mediated by I*κ*B kinase (IKK) complex, and the exposure of the nuclear location sequences. However, *R. rickettsii *is also capable of directly interacting with NF-*κ*B in its inactive form in the EC cytoplasm by an unidentified bacterial protease activity [27, 28]. Several studies have also suggested a role for protein kinase C (PKC), in the activation of NF-*κ*B by infectious agents, such as rickettsiae, and other stimuli [29].

Mitogen-activated protein kinases (MAPKs) also play a critical role in signal transduction events. Three major MAPK cascades, namely, extracellular signal-regulated kinases, c-Jun-N-terminal kinases, and p38 MAPK, have been identified and characterized, and they can be activated simultaneously or independently to constitute a central regulatory mechanism that coordinates signals originating from a variety of extracellular and intracellular mediators. Activation of enzymes in the MAPKs module by proinflammatory cytokines, reactive oxygen species (ROS), and shear stress transmits signals down the cascade, resulting in phosphorylation of many proteins with substantial regulatory functions throughout the cell, that is, NF-*κ*B. *R. rickettsii* infection selectively induces activation of p38 MAPK in ECs. Activation of p38 MAPK depends on active cellular invasion by viable rickettsiae, appears to involve generation of ROS, and subsequently facilitates host-cell invasion by *R. rickettsii* [30].

#### 2.1.4. ECs Production of Cytokines

**Figure 3:**
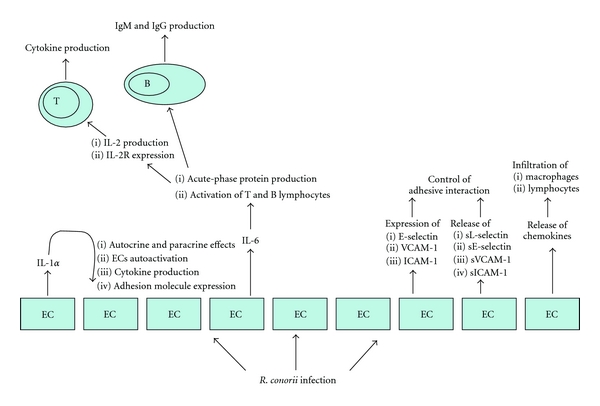
Cytokine production and induction of cellular adhesion molecules in rickettsial diseases.

Human umbilical vein ECs (HUVECs) infected by *R. conorii* actively secrete large amounts of IL-6 and IL-8 via an IL-1*α*-dependent pathway (Figure 3). Induced expression of genes encoding these proinflammatory cytokines occurs through signaling mechanisms that require activation of p38 MAPK and NF-*κ*B (see above) [31]. IL-1*α*, produced by HUVECs, remains associated with the cells, anchored to the cell surface via fatty acid acylation or through a lectin-like mechanism, mainly in a precursor form. Therefore, IL-1*α* precursor, produced by HUVECs, can be secreted and immediately bound to type I IL-1 receptor. Thus, IL-1*α* can act as an autocrine and paracrine factor on HUVECs, without being detectable in the supernatants [32]. Alternatively, IL-1*α* can be active without being secreted, fixed on endothelial surface membranes, in a cell-cell membrane interaction. IL-1*α* activates ECs, induces the production of other cytokines, such as IL-8 and IL-6, and increases the expression of adhesion molecules on both ECs and leukocytes [33]. 

IL-6 and IL-8 might play a role in the development of vasculitis induced by rickettsial infection. IL-6 may mediate acute-phase protein production associated with rickettsial infection. IL-6 might also be involved in the local differentiation and proliferation of T lymphocytes, through its stimulatory effects on IL-2 production and IL-2 receptor (IL-2R) expression on T cells and on B-lymphocytes stimulation [34]. IL-8 is a member of the chemokine family, a group of structurally related proteins with proinflammatory properties [35]. IL-8 is a powerful chemotactic agent for polymorphonuclear leukocytes, stimulates polymorphonuclear leukocyte transendothelial migration, and activates their functions, but it seems to have no effect on ECs [36]. 

#### 2.1.5. EC Production of Cellular Adhesion Molecules and Chemokines

Rickettsial infection of ECs, through the release of early-response cytokines (IL-1*α* and IL-6) and other components of the acute phase reaction, enhances the expression on the surface of ECs and polymorphonuclear leukocytes of cellular adhesion molecules (CAM), that is, E-selectin, VCAM-1, and ICAM-1, and it has been demonstrated that the enhanced rolling and the adherence of mononuclear cells to infected ECs involves E-selectin-, VCAM-1-, and ICAM-1-dependent mechanisms (Figure 3) [37]. Selectin molecules (P, L, and E), interacting with cell surface carbohydrate counterreceptors (sialyl Lewis X family), mediate rolling of the leukocytes along the vascular endothelial surface, in such a way that slows the leukocytes in the circulatory flow [38]. This permits adhesion of leukocytes to endothelial -cell-expressed adhesion molecules at the site of inflammation [39]. 

During the acute phase of boutonneuse fever (BF) (i.e., within 2 weeks after the onset of symptoms), soluble forms of these adhesion molecules (sL-selectin, sE-selectin, s-VCAM-1, and sICAM-1) are shed into plasma by proteolytic cleavage of their membrane-bound counterparts from activated leukocytes and ECs and/or by differential splicing of their mRNA. The shed forms are functionally active and could be involved in the control of adhesive interactions between cells. In particular, the increase in their levels might reduce adhesion of leukocytes and exert anti-inflammatory effects, reducing local lymphocyte and monocyte infiltration. sL-selectin and s-VCAM-1 return to the normal range in the third week, whereas sE-selectin and sICAM-1 persist at significantly high levels even after the third week of disease. Since a direct correlation between sL-selectin and fever has been demonstrated, normalization of serum levels may result in good prognosis, whereas persistence of high levels of this CAM has negative prognostic value and could be useful to monitor disease evolution. The lack of correlation among these soluble CAMs, serum secretion cytokines (i.e., IL-1*α* and IL-6), and the absolute number of leukocytes can partly be explained by the observation that the effects of these cytokines on cell surface CAM expression do not parallel the soluble CAM production, and the absolute number of leukocytes depends on the variable sources of these soluble molecules, which, in turn, bind different cell types. Differing from other CAMs, levels of sE-selectin directly correlate with the number of circulating neutrophils. Therefore, sE-selectin might be an important factor in reducing the quantity of local neutrophils [37].

Recently, a greater inflammatory EC response to *R. conorii* than that to *R. africae *was observed. These are, respectively, the etiologic agents of severe (BF) and mild (African tick bite fever, ATBF) forms of spotted fever group rickettsioses, characterized by marked increase in IL-8, MCP-1, and adhesion molecules in EC, involving Toll-like receptor 4 (TLR4) activation (see below). Importantly, there is evidence of a similar pattern *in vivo*, with increased serum levels of IL-8, VCAM-1, and E-selectin in patients with BF as compared with those with ATBF. These findings suggest a greater *R. conorii* inflammatory potential in ECs than in infection with *R. africae* related to the more severe systemic inflammation and clinical disease in the former rickettsial infection [40].

Finally, in experimental models of *R. conorii *infection of endothelium, there is prominent expression of chemokines CCL2, CCL3, CCL4, CCL12, CX3CL1, CXCL1, CXCL9, CXCL10, and Regulated by Activation, Normal T-cell Expressed and Secreted (RANTES). In particular, increased expression of CXCL1 appears to coincide with infiltration of macrophages into *R. conorii*-infected tissues, whereas expression of CXCL9 and CXCL10, known to target activated T cells through CXCR3, is significantly increased in mice infected with *R. conorii*. Rickettsiae-triggered activation of p38 MAPK and NF-*κ*B may trigger most of these responses [41].

#### 2.1.6. ECs Intracellular Killing of Rickettsiae

Perivascular infiltrations of CD4+ and CD8+ T lymphocytes, natural killer cells, macrophages, marginated elements of blood, and infected ECs themselves secrete cytokines and chemokines that activate infected ECs, macrophages, and hepatocytes, by paracrine and autocrine stimulation, to kill intracellular rickettsiae [12]. Therefore, the mechanism of killing of *R. conorii* within human target cells, mainly endothelium, and, to a lesser extent, macrophages and hepatocytes, has not been fully determined. Human cells might be capable of controlling rickettsial infections intracellularly, the most relevant location in these infections, by a cytokine-, chemokine- (i.e., RANTES), and a nitric oxide-dependent mechanism, and, in particular, by one or a combination of three different possibilities involving, (1) nitric oxide synthesis, (2) hydrogen peroxide production, (3) tryptophan degradation [42, 43].

AKN-1 cells (human hepatocytes) stimulated by IFN-*γ*, TNF-*α*, and RANTES killed intracellular rickettsiae by inducible nitric oxide synthase (iNOS) expression and nitric oxide-dependent mechanism. HUVEC, when stimulated with the same concentrations of cytokines and RANTES, differed in their capacity to kill rickettsiae by a nitric oxide-dependent mechanism and in quantity of nitric oxide synthesized. Hydrogen-peroxide-dependent intracellular killing of *R. conorii* was demonstrated in HUVECs, THP-1 cells (human macrophages), and human peripheral blood monocytes activated by cytokines. Rickettsial killing in a human macrophage cell line was also mediated by a limitation of the availability of tryptophan in association with expression of the tryptophan-degrading enzyme indoleamine 2, 3-dioxygenase (IDO), resulting in its intracellular depletion and starvation for the bacteria [44]. 

#### 2.1.7. Regulation of Programmed EC Death 

While apoptosis of infected cells is an important host defense mechanism for limiting spreading of infection, viruses and intracellular bacteria employ a variety of strategies to inhibit the host cell's apoptotic machinery to ensure and enhance the survival of the host, as a cell that dies upon infection is a cell that does not provide a niche for the intracellular pathogen's multiplication. The first evidence supporting this behaviour was demonstration that the antiapoptotic functions of NF-*κ*B are essential for survival of ECs during *R. rickettsii *infection [45]. Infection of cultured human ECs with *R. rickettsii *with simultaneous inhibition of NF-*κ*B induces activation of apical caspases-8 and -9 and also of executioner enzyme, caspase-3. The peak activity of caspase-3 coincides with the cleavage of poly-(ADP-ribose)-polymerase, followed by DNA fragmentation, mitochondrial damage, and, finally, apoptosis. Thus, activation of NF-*κ*B is required for the maintenance of mitochondrial integrity of host cells and protects against infection-induced apoptotic death by preventing activation of the caspase-8- and -9-mediated pathways [46]. The B-cell lymphoma-2 (Bcl-2) family of proteins, which includes both pro- and antiapoptotic factors, plays a critical role in regulation of apoptotic cell death by controlling mitochondrial permeability [47]. Determination of the effects of NF-*κ*B on the inhibition of proteins of the Bcl-2 family during *in vitro *EC infection by *R. rickettsii *further reveals significant changes in expression of various pro- and antiapoptotic proteins, the ultimate outcome of which is an “equilibrium shift” towards inhibition of apoptosis [48]. 

#### 2.1.8. Heterogeneity of Macrovascular and Microvascular ECs Response to Rickettsial Infection

The recently emerging concept of “EC heterogeneity” dictates that, depending on their location and physiological functions, vascular endothelium of different organ systems displays significant differences in its biological properties and activation patterns in response to known stressors. In this contest, recent studies have provided evidence for relatively similar levels of infectivity and activation of both NF-*κ*B and p38 MAPK, as common signaling mechanisms, and HO-1 mRNA expression in different types of macro- (e.g., pulmonary artery cells) and microvascular (e.g., human dermal, brain, and pulmonary endothelium) ECs infected with *R. rickettsii*. Although systematic comparative analysis of mechanisms underlying hosts cell's transcriptional activation illustrates activation of NF-*κ*B and p38 MAPK in all microvascular cell types tested, that is, human dermal, brain, and pulmonary endothelium, the most striking changes in terms of intensity of *R. rickettsii*-induced responses were observed in dermal ECs. Correspondingly, infected ECs were also found to secrete the largest amounts of IL-8 and MCP-1. Interestingly, refractoriness of brain-derived microvascular endothelium to secrete chemokines in response to infection was also revealed despite evidence for the activation of NF-*κ*B and p38 upstream signalling mechanisms and reduced ability of microvascular ECs to induce COX-2 expression and, consequently, secretion of PGE2 in response to *R. rickettsii *infection. In contrast, *R. rickettsii *infection of large vessel ECs, that is, those isolated from human pulmonary artery, produces significant amounts of PGE2, which may be attributable to increased activity of COX-2. Absence of any change in COX-2 activity during microvascular *R. rickettsii *infection suggests that the observed alterations in vascular permeability of cerebral ECs (i.e., cerebral edema) apparently involve PG-independent mechanisms [49]. 

### 2.2. Platelet and Hemostatic/Fibrinolytic Systems' Activation

In the acute phase of BF, by measurements of a major metabolite of thromboxane (TX) in the urine (11-dehydro-TXB2), a marker of platelet activation and of plasma prothrombin fragment 1 + 2, the levels of which reflect activation of prothrombin to thrombin; the occurrence of TXA2-dependent platelet activation and thrombin generation has been demonstrated *in vivo*. These phenomena could, at least partly, reflect the clinical manifestations of BF, such as vasculitis and focal microthrombus formation [50]. 

In addition, recently, a putative biochemical link has been demonstrated between endothelial dysfunction and platelet activation by investigating *in vivo* the generation of F2-isoprostanes. This family of bioactive iso-eicosanoids is produced from arachidonic acid through a process of nonenzymatic free-radical-catalyzed lipid peroxidation, and their biological activities may transduce the effect of oxidant stress into specialized forms of cellular activation [51]. Oxidative stress, induced in ECs by *R. conorii*, with increased lipid peroxidation, as shown by increased generation of F2-isoprostanes, causes endothelial dysfunction, as demonstrated by enhanced ADMA (asymmetric dimethylarginine) [52] levels. 

ADMA is an endogenous inhibitor of NO synthase, able to cause reduced NO bioavailability and, thus, endothelial dysfunction. The close relationship between production of F2-isoprostanes and ADMA levels seems to confirm the hypothesis that *R. conorii* infection, inducing oxidative stress, could, in turn, provoke endothelial dysfunction [53]. 

Furthermore, endothelial activation, induced by rickettsial infection, associated with systemic inflammation can induce platelet activation with consequent CD40L release, as demonstrated in African tick bite fever, by *R. africae*, and in BF [52, 54]. CD40L is a transmembrane glycoprotein, belonging to the tumor necrosis factor family, originally identified on CD4+ T cells, but also found in platelets. It is estimated that >95% of the circulating CD40L is derived from platelets. Binding of soluble CD40L (sCD40L) to various vascular cells (e.g., ECs or monocytes) contributes to the pathogenesis of inflammatory and thrombotic processes, as a further inflammatory stimulus to endothelial activation. Thus, once established, enhanced sCD40L shedding from platelets may sustain a vicious circle, persisting even after recovery, at a time when clinical manifestations of disease and the systemic inflammatory reaction apparently had waned [55, 56]. 

Rickettsial diseases are generally also associated with significant changes in the levels of hemostatic/fibrinolytic proteins, demonstrated by pronounced alterations in plasma concentrations of coagulation factors, natural anticoagulants, and components of the fibrinolytic system. An elevation in levels of plasma fibrin(ogen)-degradation products during *R. rickettsii* and *R. conorii* infection of humans indicates activation of the fibrinolytic system, as increased levels of circulating fibrinogen likely reflects enhanced synthesis of this acute-phase protein. The presence of circulating C1-inhibitor-kallikrein complex and lower concentrations of prekallikrein indicate activation of the kallikrein-kinin system during spotted fever rickettsioses. Rickettsia-infected ECs acquire a procoagulant phenotype. Rickettsial infection induces expression of tissue factor, secretion of plasminogen activator inhibitor, production of platelet-activating factor, expression of thrombomodulin, and release of von Willebrand factor. However, disseminated intravascular coagulation occurs rarely even in lethal cases and is not a common feature of rickettsioses [57].

### 2.3. Acute Phase Response

**Figure 4:**
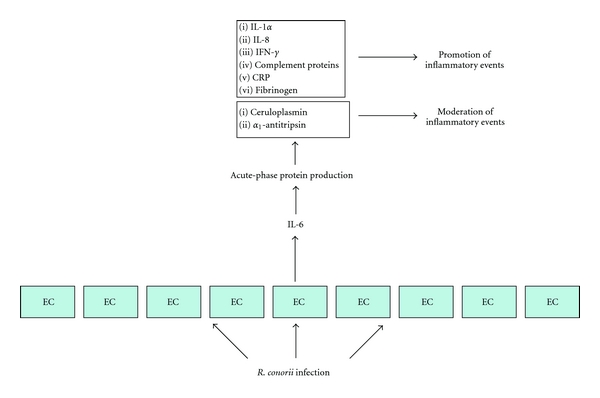
Acute phase response in rickettsial diseases.

In BF, invasion and proliferation of rickettsiae in ECs are the events responsible for destruction of vessels and for acute-phase response activation. Some acute-phase responses appear to be involved in the promotion of inflammatory events (IL-1*α*, IL-8, IFN-*γ*, complement proteins, C-reactive protein (CRP), and fibrinogen), while others appear to moderate them (ceruloplasmin and *α*1-antitrypsin). After the resolution of infection, all mediators return to the normal range, acute-phase response subsides, and the homeostatic balance is restored (Figure 4). IL-1*α* is not detectable in the blood of acute BF patients, probably because stimulation of ECs causes the production of IL-1*α* predominantly in a cell-associated form, thus contributing to the localized procoagulant and inflammatory responses which occur during rickettsial disease. Undetectable serum levels of IL-8 may be due to its prevalent presence in endothelial protrusions, either in vesicles (the Weibel-Palade bodies) or on the plasma membrane, which could be responsible for neutrophil adhesion. As for IFN-*γ*, an increase has been detected in the first week, and it offers a good explanation of the complement data. In fact, IFN-*γ* is the main inducer of C4 gene expression, acting, at least partly, on control of its transcription. Concerning factor B, its rate of gene transcription is increased by IFN-*γ*, whereas IL-1 and IL-6 act synergistically at the pretranslational level. Since in BF IL-1 is not increased and IL-6 is very high during the first two weeks of infection, IL-6 could play a role with IFN-*γ* in active synthesis of factor B in the liver. The high levels of CRP detected in the acute phase of BF could by binding cells further induce activation of the classical complement pathway. This, by generating C5a, in turn, triggers the influx of neutrophils and enhances the phagocytosis of the cells that have bound CRP and complement, thereby enhancing inflammation and tissue damage. On the other hand, CRP and complement could prove useful for elimination of infected, damaged or compromised cells, exerting anti-inflammatory activities. The increase in fibrinogen levels, in the first week of BF infection, constitutes the primary event inducing the local activation of haemostasis, through platelet activation, and it is a cursory indicator of inflammation in most human infections. The strong release of ceruloplasmin could minimize the inflammatory response, acting as a scavenger of oxygen free radicals produced by macrophages, neutrophils, and ECs. In fact, ceruloplasmin, in BF patients, returns to a baseline level nearly contemporaneously with the normalization of the white blood cell counts. *α*1-antitrypsin persists at high levels, in BF patients, until the third week, and this persistence could indicate that this proteinase inhibitor is required for a longer time, both to neutralize lysosomal hydrolases released by infiltrated macrophages and neutrophils and, more importantly, to induce release of anti-inflammatory cytokines. However, enhanced levels of ceruloplasmin, *α*1-antitrypsin, and, in general, plasma *α* fraction proteins have also been observed in several parasitic diseases, even though there was no relationship between their changes and disease evolution [58, 59].

### 2.4. Immunologic Transition from Innate to Acquired Immunity

#### 2.4.1. Toll-Like Receptors, Dendritic Cells, and the Innate Immune System

One of the mechanisms by which the innate immune system senses the invasion of pathogenic microorganisms is through pattern-recognition receptors (PRRs), which are germ-line-encoded receptors, including transmembrane Toll-like receptors (TLRs), and cytosolic nucleotide-binding oligomerisation-domain-(NOD-) like receptor (NLR) family proteins [48, 49]. Recognition of pathogen-associated molecular patterns by TLRs, either alone or as heterodimers with other TLR or non-TLR receptors, triggers signals which are responsible for activation of genes important for an effective host defense, especially those of inflammatory cytokines [60, 61]. There are at least 10 TLRs in humans. TLR signalling is mediated via interactions with adaptor proteins, including MyD88 and toll-receptor-associated activator of interferon (TRIF) [62]. Toll-like receptor 4 (TLR4) has an important role in inflammation and immunity, and its expression has been reported in most tissues of the body. TLR4 is well known as receptor for LPS, and its activation is critical for response to gram-negative bacteria. Recently, mutations in human TLR4 were found to be associated with hyporesponsiveness to LPS and increased risk of gram-negative infections both in humans and experimental animals [63]. The +896 A→G nonsynonymous single-nucleotide polymorphism of the TLR4 gene, causing the Asp299→Gly change in the extracellular domain of TLR4, attenuates receptor signalling, decreases endotoxin responsiveness, and determines poor outcomes from sepsis [64]. Interestingly, it has been demonstrated that the +869G single-nucleotide polymorphism of the TLR4 gene is overexpressed in BF patients with significant differences in the frequency of TLR genotypes and alleles between BF patients and age-matched controls. These data might be interpreted as one hypothetical basis for genetic susceptibility to BF [65]. 

The role played by dendritic cells (DCs), key initiators and orchestrators of the immune response, is important to understand in rickettsial infections. TLR4 signalling, due to ligation with rickettsial LPS, is important in activating antigen-presenting cells, for example, DCs, toward production of proinflammatory cytokines (i.e., IL-2, IL-6, IL-12, and IL-23), initiation of antirickettsial innate immunity (namely, expansion of the natural killer (NK) cell population and subsequent production of IFN-*γ*) and production of adaptive T_H_1 type or T_H_17 responses (which play a critical role in autoimmunity and are also implicated in protective immune responses to bacterial pathogens). Conversely, lack of TLR4 stimulation during antigen presentation leads to disproportionate expansion of CD4+ CD25+ Foxp3- T-regulatory lymphocytes (T_reg_) and subsequent suppression of proinflammatory immune responses to rickettsiae. C3H/HeJ mice, which are naturally defective in TLR4 signaling, develop overwhelming, fatal rickettsial infections when given an inoculum that is nonfatal for TLR4-competent mice. C3H/HeJ mice have a reduced T_H_1-type response and a significantly greater percentage of T_reg_ cells in their peripheral lymph nodes, which could potentially suppress proinflammatory responses by production of IL-10 or transforming growth factor-*β* (TGF-*β*), and limit the initiation of adaptive immunity, by decreasing the number of effector cells during antigen stimulation (see below). In contrast, mice with competent TLR4 responses are more resistant to rickettsial infection. Moreover, this resistance is associated with an increase in number of known antirickettsial effector cells, specifically T_H_1- and T_H_17-polarized CD4+ and CD8+ T lymphocytes [66, 67].

TLR4-ligated DCs induce recruitment of NK cells to draining lymph nodes. Furthermore, this recruitment and production of NK cell-derived IFN-*γ* in draining lymph nodes are important in augmenting the T_H_1 immune response. It has been documented that TLR4 stimulation is also important in inducing NK cell activation* in vivo*. The number and percentage of NK cells in spleen are consistently lower in C3H/HeJ mice than in TLR4-competent ones after *R. conorii *infection. Additionally, splenocytes from C3H/HeJ mice have less NK cell cytotoxic activity *in vitro* than those from TLR4-competent mice. Moreover, TLR4-competent mice have significantly higher serum levels of IFN-*γ* during early infection, and subsequent investigation demonstrated that NK cells in TLR4-competent mice produce significantly more IFN-*γ* after *in vitro* stimulation. This IFN-*γ* production is important in inducing early NO production in infected ECs [68].

Taken together, all these data reveal an important role for DCs in recognizing rickettsiae through TLR4 and that a vigorous proinflammatory response induced by DCs is associated with protective immunity to rickettsiae. Subsequently, generation of antigen-specific immunity is crucial to complete protection [66–68].

#### 2.4.2. CD4+ Lymphocytes and Related Cytokines

In acute BF patients, a reduction of circulating T cells, and, in particular, of CD4+ (helper/inducer T cells), CD4+/CD45RO+ (memory T cells), and CD4+/CD45+ (naive cells) subsets have been demonstrated. These modifications could be due to cell adhesion to vascular endothelium, followed by their entrance into the sites of inflammation. Apoptosis, spontaneous and/or activation-induced, could also have a role in this diminution [69, 70]. Other lymphocytic subsets (CD8+ (suppressor/cytotoxic T cells), CD16+ (NK cells), and CD20+ [B cells)) tend to decrease but not statistically significantly, whereas monocytes (CD14+/HLA-DR+) are significantly increased. All cell subsets return to normal levels after successful treatment except for monocytes, which persist at a high level after recovery. Inflammatory and immunologic responses, mediated by increased levels of T_H_1-type (TNF-*α* and IFN-*γ*) and T_H_2-type cytokines (IL-10 and IL-6), appear to be important for recovery from infection. In detail, serum levels of TNF-*α*, IFN-*γ*, IL-10, and IL-6 are significantly increased in patients with acute BF, compared with those from control healthy subjects. On the contrary, *in vitro* peripheral mononuclear cells from acute BF patients produce low levels of IFN-*γ* and IL-10 in response to mitogen or specific antigen, whereas TNF-*α* and IL-6 responses are in the normal range. It is possible that IFN-*γ* and IL-10 are produced at the sites of infection, as observed in leishmaniasis, as a result of local recruitment and accumulation of activated CD4+ T cells in the perivascular area adjacent to infected ECs. Furthermore this observation is compatible with observed reduction of circulating CD4+ cells. In contrast, normal production of TNF-*α* and IL-6 could be due to the presence of high levels of peripheral monocytes, important sources of TNF-*α* and IL-6 production. Dramatic increase in TNF-*α* in the first week after onset of symptoms, together with IFN-*γ*, is one of the early events observed in acutely ill BF patients, followed by increases of IL-10 and IL-6. IFN-*γ* levels drop sharply in the second week after onset of fever, whereas TNF-*α*, IL-10, and IL-6 gradually decline, reaching normal levels after the third week [69, 71]. 

**Figure 5:**
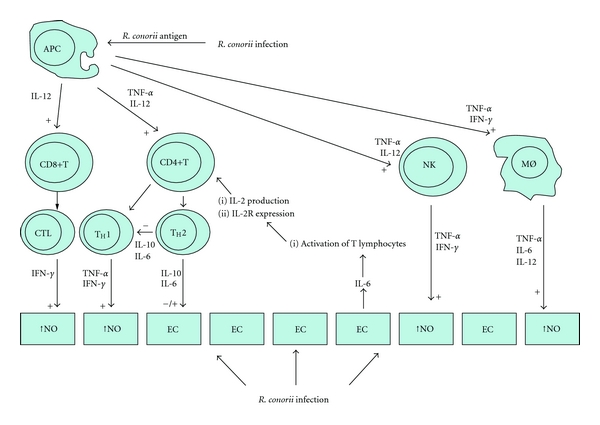
Immunologic alterations in rickettsial diseases.

Antigen-presenting cells activated by rickettsial antigens produce high levels of TNF-*α* and IFN-*γ*. TNF-*α* induces TNF-*α* and IFN-*γ* production from activated CD4+ T cells with T_H_1 phenotype and NK cells. One important effect of TNF-*α* and IFN-*γ* on macrophages is induction of receptors for TNF-*α*, so the binding of TNF-*α* on macrophages might further activate these cells, by an autocrine TNF-*α* loop, thus increasing their antirickettsial activity via induction of NO synthesis (Figure 5) [72–74]. Persistence of high serum levels of TNF-*α* in recovered patients could be a sign of residual local lesions. Patients with severe forms of BF, associated with disseminated intravascular coagulation, had very high levels of TNF-*α* that persisted for a long time, and decreased only when the condition of the patients improved. A relationship has also been observed between serum TNF-*α* and a high level of production of one of two soluble TNF receptors, persisting high values of sTNF-RI in BF could be an indicator of disease activity [69, 75].

Because IL-10 (a T_H_2-type cytokine) suppresses the ability of IFN-*γ*-activated macrophages to produce inflammatory mediators, its persistent high levels could be owing to its ability to downregulate the potentially tissue-damaging effects of responses mediated by increased T_H_1-type cytokines [76, 77]. 

The profile of serum IL-6 (another T_H_2-type cytokine) production in patients with acute BF and after recovery is similar to that of IL-10. In acute BF, high IL-6 levels could be due to synthesis at sites of infection by activated and damaged ECs, infiltrating monocytes, and T_H_2 T cells. In acute BF, IL-6 could act as an inflammatory cytokine, rather than as a growth and differentiation factor for B cells and immunoglobulin production. The fact that B cells are reduced in acute BF in the face of high levels of IL-6 is consistent with this hypothesis [44, 69]. Later, when IL-10 begins to inhibit T_H_1 cells that produce TNF-*α* and IFN-*γ*, favouring proliferation and secretion of T_H_2 cytokines, such as IL-6, terminal differentiation of B cells to immunoglobulin-producing cells could be favoured [69, 78]. In agreement with this scenario, there is normalization of B cells in recovered patients. The specific antibody titer increases only 2 weeks after infection and peaks in 4 weeks. Afterwards, IgM decreases, and IgG remains high for several months [69, 79]. Since soluble IL-6R (sIL-6R*α*) could enhance rather than inhibit the biological activity of IL-6 both *in vitro* and *in vivo*, the elevated levels of sIL-6R*α* detected in the first weeks of infection in BF patients might result in increased IL-6 activity. In this case, IL-6-sIL-6R*α* complexes could act as strong activators of the inflammatory response [69, 80].

Therefore, in BF patients, T_H_1- and T_H_2-type responses are not characteristically polarised, as both activating (i.e., TNF-*α*, IFN-*γ*) and suppressive (i.e., IL-10 and IL-6) cytokines are detected [81, 82]. It is possible that in BF, as in any other inflammatory environment, counterbalancing mechanisms are normally produced to curtail the process. IL-10 and IL-6 are capable of derailing T_H_1-type responses and deactivating macrophages, thereby moderating tissue injury [83].

IL-12, a cytokine produced by B cells, phagocytic cells, and other antigen-presenting cell types, induces cytokine production, primarily IFN-*γ*, from T cells with T_H_1 phenotype and NK cells, acts as a growth factor for activated T cells and NK cells, enhances the cytotoxic activity of NK cells, and favours cytotoxic T-lymphocyte generation, thus exerting stimulatory effects on T_H_1-type responses and downregulating T_H_2-type activity [84]. In BF patients, IL-12, predominantly produced by monocytes, appears to act very early, favouring T_H_1 differentiation and T_H_1-type cytokine production and contributing to phagocytic cell activation and innate resistance to intracellular pathogens. Therefore, IL-12 could, if produced in effective amounts, favour rickettsial clearance, activating the T_H_1 responses, as observed in other intracellular infections [84, 85].

Finally, some authors have described the occurrence in patients with BF of haemophagocytic lymphohistiocytosis (or haemophagocytic syndrome), a potentially fatal hyperinflammatory syndrome, characterized by histiocyte proliferation and haemophagocytosis. The pathophysiology of haemophagocytic lymphohistiocytosis in BF patients is not fully understood. However, an uncontrolled immune response can lead to hypersecretion of cytokines, upregulation of adhesion molecules and MHC I and II molecules on monocytes-macrophages, and expansion of inflammatory monocytes, together with impaired or absent function of NK cells and cytotoxic T cells. This exaggerated inflammatory response could result in uncontrolled proliferation and phagocytic activity of histiocytes [86].

#### 2.4.3. Role of CD8+ T Cells

CD8+ T lymphocytes, perhaps activated by antigen-presenting ECs, in association with antigens on class I of major histocompatibility complex (MHC-I), contribute to protective immunity to rickettsiae by both MHC-I-restricted cytotoxic T-lymphocyte (CTL) activity and production of IFN-*γ*. However, CTL activity might be more critical to recovery from rickettsial infection than the effects of IFN-*γ* production by CD8+ T cells (Figure 5). Therefore, CD4+ and CD8+ T lymphocytes are both a potentially rich source of IFN-*γ*, which could activate endothelial rickettsicidal activity and tip the balance in favour of survival. However, in mice, depletion of CD4+ cells has no observed effect on course or outcome of infection. In contrast, CD8+-depleted mice, infected with ordinarily sublethal dose of *R. conorii*, remain persistently infected and ill, and a high proportion of these animals die of uncontrolled rickettsial infection and its consequent pathologic effects. Rickettsia-infected ECs, and to a lesser extent, rickettsia-infected macrophages, are the targets of MHC-I-restricted CTL-mediated clearance of the infection. Perforin is one of the mechanisms of elimination of *R. conorii*-infected cells, but it is probably not the only one. As of yet, the roles of Fas-Fas ligand interaction and/or granulysin, in antirickettsial CTL activity, might also be important [87, 88]. 

#### 2.4.4. Other Markers of Immunologic Activation

The strong T-cell activation, during BF, is also attested by observation of a very significant release of the *α*-chain of high-affinity IL-2R, in soluble form (s-IL2R), in serum and urine of acute BF patients [89]. IL-2R is the main cellular mediator of the actions of IL-2, as a growth and differentiation factor for T, B, and NK cells and as an activator for macrophages. sIL-2R, released both *in vitro* and *in vivo* following lymphocyte activation, binds IL-2, and, thus, it potentially downregulates IL-2-driven proliferation and IL-2-dependent cell-mediated responses [90]. sIL-2R is significantly increased in acute BF and returns to normal when patients recover from disease. Serum levels of sIL-2R correlate positively with those in urine, indicating that sIL-2R undergoes glomerular filtration [89]. 

Other markers produced by functionally active lymphocytes such as soluble CD4 (sCD4), soluble CD8 (sCD8), *β*2-microglobulin (*β*2-M), and soluble class I antigens (sHLA-I) are released, in the sera of patients with acute BF, and correlate with the evolution of infection [91]. The pathophysiological functions of these molecules are not known. It is likely that sHLA-I, shed as dimers with *β*2-M, contains the peptide of foreign rickettsial antigen within the groove, and in such a way it can potentially recognize reactive T cells. Thus, sHLA-I could play a relevant role in regulating the immune response, being released by activation and contributing itself to activation of other cells [92, 93]. 

#### 2.4.5. Role of CD4+ CD25+ Foxp3- T Regulatory (T_reg_) Lymphocytes 

Acute severe human rickettsial diseases have been characterized as diseases that stimulate a dominant type 1 immunity and unresponsiveness or suppression of CD4+ T cells, with transient immune dysregulation. There is a suppressed CD4+ T_H_1-cell response during lethal rickettsial infection in susceptible C3H/HeN mice (that mimic the pathogenesis of severe human rickettsial infection), including inhibition of IFN-*γ* production by spleen cells in response to rickettsial stimulation compared to production in sublethally infected mice, a serum level of IFN-*γ* significantly lower than that in sublethally infected mice, suppressed or unresponsive proliferation of CD4+ T cells in response to anti-CD3 or specific antigen stimulation, inhibition of IL-2 production by splenocytes, and, finally, a lower frequency of antigen-specific IFN-*γ*-producing CD4+ T cells than that in sublethally infected mice. Immunosuppression induced by a high dose of rickettsiae may account for the uncontrolled bacterial burden and overwhelming infection. The suppressed immune response observed in mice infected with a lethal dose of *R. conorii *is associated with substantial, significantly greater expansion of T_reg_ cells in the infection sites. Therefore, generation of T_reg_ cells might be a potential mechanism contributing to host susceptibility to acute severe rickettsiosis. In particular, it has been demonstrated that DCs from resistant C57BL/6 mice exhibit higher expression levels of MHC class II and higher IL-12 production upon rickettsial infection and are more potent in priming naïve CD4+ T cells to produce IFN-*γ*, whereas DCs from susceptible lethally infected C3H mice promote the induction and expansion of a novel phenotype of suppressive CD4+ CD25+ T-bet^−^ Foxp3^−^ CTLA-4^high^ T-reg cells, consisting of both IL-10-producing adaptive T_reg_ cells and IFN-*γ*-producing T-effector cells. This novel T_H_1-cell-related adaptive T_reg_-cell population contributes greatly to deep immunosuppression during acute severe rickettsiosis via as-yet-unidentified mechanisms that may involve IL-10 production, or CTLA-4 (cytotoxic T-lymphocyte antigen 4) function, or an indirect process via an influence on DCs. It is also possible that IL-10-producing antigen-presenting cells, such as DCs, are involved in immunosuppressive phenomena in addition to the suppression mediated by T_reg_ cells and that interactions between DCs and T_reg_ cells, rather than T_reg_ cells alone, may play a crucial role in the balance between an efficient immune response and tolerance/susceptibility [94, 95].

#### 2.4.6. Intralesion Production of Cytokines and Chemokines in BF

Very few human studies directly examined either the local mediators of inflammation or the immune response at the site of infection (e.g., skin) in rickettsial diseases. Skin-biopsy samples from patients with BF, collected 3–14 days after onset of fever, revealed a balanced mixed type 1, pro-inflammatory and anti-inflammatory responses, as reflected by elevated levels of mRNA expression of TNF-*α*, IFN-*γ*, and IL-10. Increased levels of IL-10 suggest the presence of an immunoregulatory mechanism that can help to avoid severe tissue damage due to excess intralesional production of proinflammatory type 1 cytokines. There is also a positive correlation between high levels of IFN-*γ* mRNA and TNF-*α* mRNA and the production of enzymes involved in limiting microbial growth (i.e., iNOS and IDO). Finally, although the median mRNA-expression levels of IFN-*γ*, TNF-*α*, IL-10, iNOS, and IDO were not statistically significantly different between patients with mild, moderate, and severe MSF, there was a trend toward expression of substantially higher levels of certain biomolecules in either mild or severe MSF. In detail, patients with severe BF showed higher levels of TNF-*α* mRNA and lower levels of IFN-*γ* mRNA compared with those of mild BF. All of the patients with severe BF had higher IDO and RANTES mRNA-expression levels and lower levels of iNOS mRNA compared with those of mild BF. The level of IL-10 mRNA expression was comparable in all 3 groups of patients. Therefore, IFN-*γ* is critical for protection against severe BF, and protection is dependent on the expression of iNOS mRNA, whereas TNF-*α* and RANTES may play a dual role, being involved in both protective antirickettsial immunity and the pathogenesis of severe BF [96]. 

## 3. Orientia tsutsugamushi

*Orientia tsutsugamushi* infects predominantly ECs, even though it may be found in several other cells, including DCs, macrophages, polymorphonuclear leukocytes (PMNs), and lymphocytes. The bacterium invades host cells by induced phagocytosis and then is taken into a phagosome. Loss of phagosomal membranes is frequently observed, suggesting a mechanism for *O. tsutsugamushi* escape from phagosome into cytoplasm. The virulence factors related to phagosomal membrane lysis, however, have not been identified. Then *O. tsutsugamushi* usually propagates in host cytoplasm via binary fission. Groups of budding *Orientia* (the budding process differs from that of enveloped viruses as the host cell membrane surrounds the bacterial cell wall rather than being incorporated into the organisms' structure) are clearly observed on the cell surface after 2-3 days of incubation. The released bacterium covered by host cell membrane may either directly infect neighboring cells by fusion of membranes or the surrounding host cell membrane may be lost, leaving naked *Orientia* to invade other cells [97].

### 3.1. Invasion, Injury, and Activation of Infected Target Cells

#### 3.1.1. ECs, Dendritic Cells, and Macrophages Invasion

**Figure 6:**
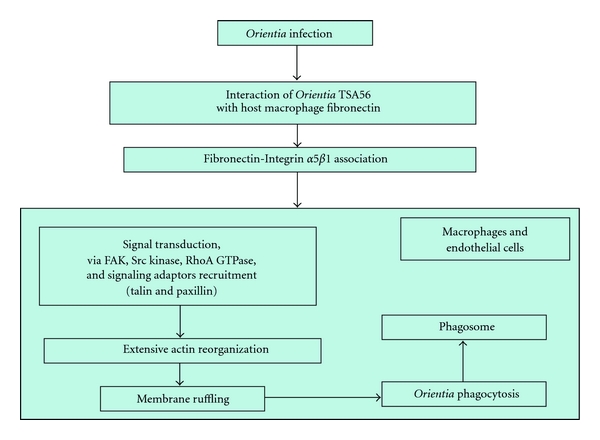
*Orientia *macrophages and endothelial cells invasion.

Bacterial invasion of host cells is primarily mediated by interactions between bacterial surface components and complementary host receptors, which stimulate host signal transduction pathways required for bacterial access. *O. tsutsugamushi *uses host fibronectin interactions with one of its outer membrane proteins, the 56-kDa type-specific antigen (TSA56), to carry out internalization. Then, fibronectin facilitates bacterial entry into the host cells via interactions with integrins, that is, *α*5*β*1. After integrin engagement, signaling molecules at the inner surface of the plasma membrane, which act as integrators of responses to extracellular stimuli, are activated. In detail, focal adhesion kinase (FAK), Src kinase, and RhoA GTPase are activated by *O. tsutsugamushi* invasion, and the signaling adaptors talin and paxillin are recruited to the site of infection. Furthermore, extensive actin reorganization and membrane ruffling in the region surrounding the *O. tsutsugamushi *cells are induced within 10 min. after attachment. Therefore, *O. tsutsugamushi *exploits integrin-mediated signaling and rearrangements of the actin cytoskeleton for invasion of eukaryotic host cells. However, many other membrane proteins, as well as TSA56 (e.g., products of *sca* genes), may interact with other receptors to trigger the observed downstream signaling events, involving recruitment of host endocytic machinery components and their specific activation, which ultimately lead to the extensive actin reorganization required for pathogen entry [98, 99] (Figure 6).

#### 3.1.2. ECs, Dendritic Cells, and Macrophages Activation

**Figure 7:**
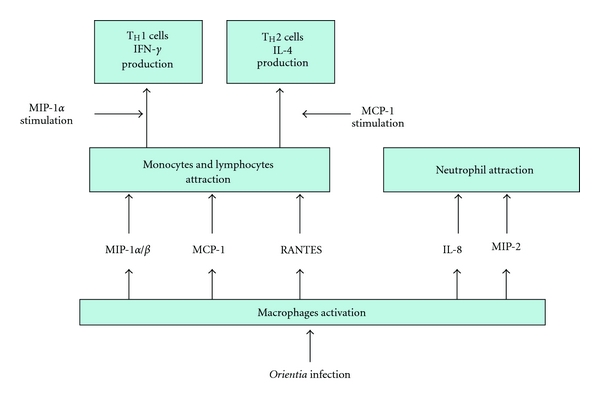
*Orientia *macrophages activation.

**Figure 8:**
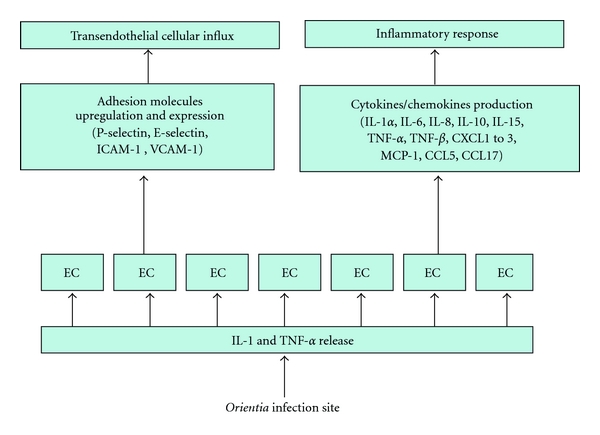
*Orientia *endothelial cells activation.

Inflammation is initiated by *O. tsutsugamushi*-infected cells in the dermis. Proinflammatory cytokines and chemokines secreted by activated DCs, that is, TNF-*α*, IL-1*3β*, IL-6, macrophage inflammatory protein (MIP)-1 *α*/*β*, MIP-2, and MCP-1, are the main ones responsible for leukocyte recruitment into inflammatory tissues. Members of the CC chemokine subfamily, which include MIP-1 *α*/*β* (CCL3/CCL4), MCP-1 (CCL2), and RANTES (CCL5), preferentially attract monocytes and lymphocytes, while those of the CXC chemokine subfamily, such as IL-8 and MIP-2 (CCL8), are potent neutrophil attractants. While CC chemokines, such as MIP-*α*/*β* and RANTES, are efficient chemoattractants for T_H_1 cells, T_H_2 cells were not attracted by them. Stimulation of T cells in the presence of MIP-1*α* enhances production of IFN-*γ* by T_H_1 cells, while stimulation of T cells in the presence of MCP-1 leads to an increase of IL-4 production (a T_H_2 cytokine). Taken together, T-cell differentiation by a subset of chemokines produced by activated DCs might be a crucial factor in the induction of a resistant versus a susceptible immune response to *Orientia* infection (Figure 7). ECs are another key participant in the inflammation process. Activation of ECs is stimulated by proinflammatory cytokines, including TNF-*α* and IL-1, which are released from infected sites and result in the upregulation of cell adhesion molecules, such as P-selectin, E-selectin, ICAM-1, and VCAM-1, to promote cellular influx via transendothelial migration, as well as production of cytokines and chemokines, such as IL-1*α*, IL-6, IL-8 (CXCL8), IL-10, IL-15, TNF-*α* and TNF-*β*, CXCL1 to 3 (Gro), MCP-1 (CCL2), CCL5, and CCL17, to initiate and propagate local inflammatory responses (Figure 8). In addition, in patients with scrub typhus, the serum levels of soluble L- and E-selectin correlate with the symptoms of disease. MIP-1 *α*/*β* (CCL3/CCL4), MCP-1 (CCL2), and RANTES (CCL5), produced by activated macrophages, are induced via NF-*κ*B activation, whereas activated ECs generate MCP-1 (CCL2) and CXCL8 (IL-8) independent of NF-*κ*B activation [100, 101].

### 3.2. Immunologic Transition from Innate to Adaptive Immunity

#### 3.2.1. Innate Immune System

**Figure 9:**
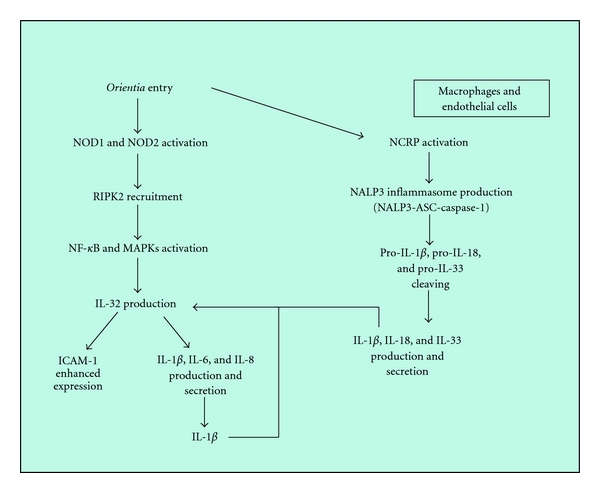
Innate immune system in oriential infection.

As TLRs are important for innate immunity to rickettsiae, NLR family proteins are involved in innate immune mechanisms to *Orientia* infection. The NLR family is composed of 22 intracellular molecular-pattern-recognition molecules. One subfamily includes NOD1 and NOD2, which sense peptidoglycan polymers from cell wall components. Activation of NOD1 and NOD2 triggers recruitment of the adapter protein receptor-interacting serine-threonine kinase 2 (RIPK2 or RIP2), followed by activation of NF-*κ*B and MAPKs. Another subfamily includes the NLRP proteins, which are essential for regulation of caspase-1 activation via the N-terminal pyrin domain by inflammasome formation, consisting of NALP3, caspase recruitment domain (ASC), and caspase-1. The NALP3 inflammasome then cleaves pro-IL-1*β*, pro-IL-18, and pro-IL-33 to mature IL-1*β*, IL-18, and IL-33, respectively. During *O. tsutsugamushi *infection, NOD1 senses an *Orientia* component in ECs and activates the downstream pathway of NF-*κ*B, which leads to production of IL-32, an IL-18-induced cytokine, previously recognized as natural killer cell transcript 4 (NK4). Such increased IL-32 levels affect secretion of proinflammatory cytokines, that is, IL-1*β*, IL-6, and IL-8, as well as ICAM-1 expression in ECs. In addition, in ECs, IL-1*β* induces IL-32 production, followed by an increase in the levels of IL-1*β*, IL-6, IL-8, and ICAM-1. Therefore, IL-32-induced IL-1*β* expression in *O. tsutsugamushi*-infected ECs may reciprocally enhance IL-32 secretion. Consequently, IL-32 and IL-1*β* might form a positive feedback loop on ECs during the process of inflammation [102] (Figure 9).

#### 3.2.2. CD4+ Lymphocytes and Related Cytokines

Type-1 cell-mediated immunity and IFN-*γ* production of T cells in response to *O. tsutsugamushi* antigen is essential for immune protection against infection, whereas the opposite T_H_2 subset is considered to be detrimental. In animal models (intraperitoneal injection of *Orientia* in mouse, not perfectly corresponding to the natural modality of human infection, via intradermal penetration), T_H_1- and T_H_2-type responses are not clearly polarised, as both activating (i.e., IL-12, IFN-*γ*) and suppressive (i.e., IL-10) cytokines are simultaneously detected. It is possible in scrub typhus, like in other infectious disease (e.g., visceral leishmaniasis), that counterbalancing mechanisms are normally produced to modulate the process and ensure homeostasis within the host. Therefore, *O. tsutsugamushi *activates both proinflammatory and modulatory pathways, via IL-10, in the early stages of infection. An early step in generating T_H_1 responses is IL-12 production by antigen-presenting cells (DCs, phagocytes, and mesothelium), which targets natural killer cells and T cells, inducing IFN-*γ* production, whereas early IL-10 production inhibits this response. IL-10 inhibits production of T_H_1 type cytokines, including IFN-*γ*, IL-1, IL-2, IL-3, GM-CSF, and TNF-*α* and thereby inhibits the development of T_H_1 immunity that is essential for clearing *O. tsutsugamushi *in infected ECs. In contrast, IFN-*γ* inhibits proliferation of T_H_2 cells, thereby inhibiting production of T_H_2-derived cytokines, including IL-4, IL-5, and IL-6 which are essential for B-cell differentiation and isotype switching [103]. 

**Figure 10:**
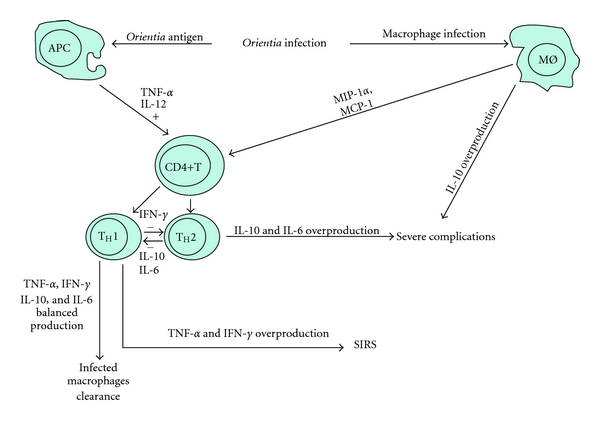
T lymphocytes and related cytokines in oriential infection.

Although the cytokine and chemokine profile demonstrates that *O. tsutsugamushi* infection results in immune reaction via soluble mediators from infected tissues or cells, this intracellular bacterium seems to exploit immune evasion mechanisms for survival. In addition, patients with tsutsugamushi disease sometimes suffer from severe clinical complications in the absence of appropriate antimicrobial chemotherapy. The exact mechanism leading to such severe complications is not well understood yet. In animal models (with the above-mentioned limitations), one of the possible mechanisms employed by *O. tsutsugamushi* involves the suppression of proinflammatory cytokine production, including TNF-*α* and IL-6, via induction of IL-10 secretion from infected macrophages and mesothelium. It has been reported that an imbalance of these two cytokines, with low levels of TNF-*α* and high levels of IL-10, may be important in worsening the condition of patients affected with bacterial infections. Alternatively, as an excessive inflammation-related cytokine response during the initial and essential host response to an infectious challenge develops, it may lead to harmful, or even fatal, consequences, as seen in septic shock or multiple organ failure, that is, SIRS. In a life-threatening infection, systemic release of several proinflammatory cytokines is not properly regulated, and higher serum concentrations of inflammatory cytokines have been strongly implicated in the development of SIRS. Some studies demonstrated that serum levels of TNF-*α* have some relationship with the severity of tsutsugamushi disease. The TNF-*α* levels in the acute phase could predict the severity of this infectious disease. As TNF-*α* is essential for host defense, TNF-*α* underproduction may help proliferation of the pathogen, whereas its overproduction may be harmful to the host [104] (Figure 10).

#### 3.2.3. Humoral Immunity

It is unclear if anti-*Orientia* antibodies, appearing early enough in infection, really affect the course of infection, or if passive immunization only reflects what might be a benefit of vaccination. However, humoral immunity might play a role in protective immunity to *O. tsutsugamushi* by inhibiting the events required in attachment, entry, and/or trafficking and replication in the cytoplasm. It has been demonstrated that antibodies enhance opsonophagocytosis of *O. tsutsugamushi *by professional phagocytes, such as macrophages and PMN. In addition, antibodies inhibit invasion of *O. tsutsugamushi *into non-professional phagocytes, such as ECs, epithelial cells, and fibroblasts [105]. However, neither cytokines nor antibodies alone enable macrophage cultures to completely suppress oriential infection. This indicates that both cellular and humoral immunity play a role in clearing *O. tsutsugamushi *by facilitating uptake and ensuing intracytoplasmic destruction of the bacterium. Although identification of protective antigens is important to understand homotypic and heterotypic immunity to scrub typhus, the molecules that play important roles in generating protective immunity are partially known. Antigenic heterogeneity among *O. tsutsugamushi* strains is so pronounced that whole cell vaccines prepared against one strain generally fail to protect mice against infection from others. *Orientia* lacks both peptidoglycan and LPS, containing the major strain-variable 56-kDa protein, as well as the antigenically variable 110-, 47- and 25-kDa proteins. Among all antigens, the 56-kDa protein is often recognized by both human and animal host immune systems during infection. It is structurally and functionally nearly identical to the eukaryotes' protein known as Heat Shock Protein (Hsp) 60. There is substantial evidence indicating that heat shock proteins mainly serve as target molecules in the anti-infectious or autoaggressive immune responses. For these reason, the 58-kDa protein should be carefully considered as a recombinant vaccine candidate [106]. 

## 4. Ehrlichiosis

### 4.1. Target Cells

*E. chaffeensis* is confined to cytoplasmic membrane-bound vacuoles within monocytes/macrophages and DCs, replicating to form microcolonies, called *morulae*, that contain one to over 400 organisms. Morphologically, individual ehrlichiae are coccoid and coccobacillary and exhibit two ultrastructural cell types: a larger reticulate and a smaller dense-cored cell. *Ehrlichia* reticulate cells and dense-cored cells represent the bacterial replicating and infectious forms, respectively. The dense-cored cell form of *E. chaffeensis* binds to the host cell surface where it is rapidly internalised and completes the developmental cycle within 72 h [107].

### 4.2. Invasion, Injury, and Activation of Target Cells

#### 4.2.1. Monocyte/Macrophage and Dendritic Cell Invasion

The presence of long-period tandem repeats distributed in intergenic regions of *Ehrlichia* is well recognised. They have also been found in a small group of ehrlichial proteins, named “tandem repeat proteins” (TRPs), that is, TRP120 and TRP47, associated with host-pathogen interactions, such as adhesion, internalisation, actin nucleation, and immune evasion [108]. 

**Figure 11:**
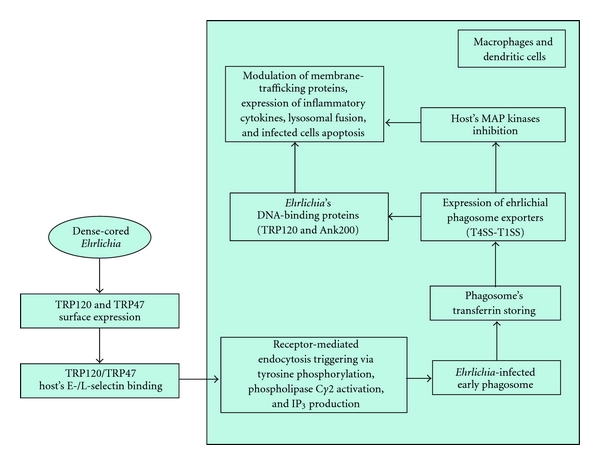
Ehrlichial monocytes/macrophages and dendritic cells invasion.

Infection of the host cell involves dense-cored ehrlichiae that express TRP120 on the surface. It has important role in the binding and entry process, and a potential role has also been demonstrated for TRP47. Ehrlichiae binding to the host cell occurs through membrane receptors, such as E- and L-selectin and other glycosylphosphatidylinositol-anchored proteins located in caveolae (tiny indentations in the cell surface membrane), triggering receptor-mediated endocytosis that involves signalling transduction events, including transglutamination, tyrosine phosphorylation, phospholipase C*γ*2 activation, inositol-(1,4,5)-trisphosphate (IP3)- production and increases in the intracellular calcium concentration. The vacuoles in which the organism enters contain caveolin-1, GM1 ganglioside, and phospholipase C*γ*2. Later, after infection, vacuoles containing replicating ehrlichiae show the characteristics of early endosomes (i.e., the presence of Rab5A, early endosomal antigen 1 (EEA1), and vacuolar (H+) ATPase, together with vesicle-associated membrane protein 2, major histocompatibility class II and *β*2-microglobulin) and accumulate transferrin and its receptor. As a matter of fact, *Ehrlichia* survival depends on an available supply of intracellular iron, and the antiehrlichial activity of IFN-*γ* is mediated by limiting cytoplasmic iron availability (see below) [109–111] (Figure 11). 

#### 4.2.2. Modulation of Host Cell Gene Expression

*Ehrlichia chaffeensis* seems to actively modulate host cell gene transcription and function despite the fact that the bacterium develops exclusively inside a vacuolar inclusion, separated from host cell cytosol by a host membrane. Therefore, host cell gene transcription modulation is related to secretion of specific ehrlichial effector proteins into the host cell cytosol, which requires active secretion systems. Such delivery mechanisms have been identified and include many of the known type IV secretion system (T4SS) components; Sec-dependent and Sec-independent protein export pathways for protein secretion across the membrane, as well as a putative type I secretion system (T1SS), have also been identified. A consequence of effector proteins-host-cell interaction is downregulation of transcription factors and transcription of target genes related to host defence and other cellular functions, at least in part by inhibition of host mitogen-activated protein (MAP) kinases. Discovery of *Ehrlichia* DNA-binding proteins (i.e., TRP120 and Ank200) provides another mechanism by which host cell gene transcription can be directly modulated. Specific cellular processes which appear to be modulated are membrane-trafficking proteins, expression of proinflammatory cytokines, and infected cell apoptosis [112, 113]. 

*Ehrlichia chaffeensis* lives in early endosome and inhibits its maturation to evade fusion with lysosomes and destruction by lysosomal enzymes. Moreover, *E. chaffeensis* inhibits transcription of genes involved in membrane trafficking and lysosomal fusion, such as Rab5, synaptosome-associated protein 23 (SNAP23), and syntaxin 16 (STX16) [114] (Figure 11).

Survival in mononuclear phagocytes requires the ability to evade the innate and adaptive immune responses (see also below*). Ehrlichia chaffeensis* inhibits transcription of cytokines involved in the early innate immune response and cell-mediated immune response to intracellular bacteria. As a matter of fact, this bacterium avoids stimulation of IL-12 production and represses IL-15 and IL-18 production; these cytokines play fundamental roles in stimulating T_H_1 and NK cells to produce IFN-*γ*, which then promotes macrophages' killing of phagocytosed bacteria. IL-12 and IL-15 also activate destruction of cells infected with intracellular bacteria by NK cells and cytotoxic T lymphocytes. Thus, repression of IL-12, IL-15, and IL-18 may help *E. chaffeensis* to evade the host's innate and adaptive immunity [109]. 

**Figure 12:**
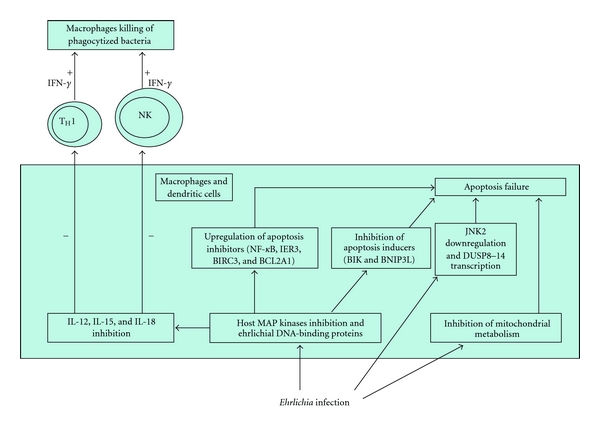
Modulation of host cell gene expression in ehrlichial infection.

Apoptosis is an innate cellular defence mechanism against microbes that is modulated by many bacterial pathogens, and there are new data that indicate that *Ehrlichia* also modulate host cell death. For most intracellular pathogens, apoptosis induction leads to pathogen killing and infection clearance, and this is thought to be beneficial to the host and to enhance the immune response to the infection. Delaying or preventing apoptosis could be a mechanism to enhance ehrlichial survival by preventing host cell death and subsequent immune recognition. In the case of ehrlichial infection, there seem to be several mechanisms involved in apoptosis modulation and inhibition. Modulation of genes associated with inhibition of apoptosis, cell cycle regulators, and mitochondrial function is observed early during *E. chaffeensis* infection and is probably necessary for delaying host cell death. Particularly, *E. chaffeensis* infection both upregulates transcription of apoptosis inhibitors, such as NF-*κ*B, IER3, BIRC3, and BCL2A1, and inhibits apoptosis inducers, such as BIK and BNIP3L, which initiate apoptosis by inactivating BCL2 proteins, thus impairing host cell apoptosis and maintaining a prolonged growth opportunity for ehrlichiae. At the same time, during the early infection stages, *E. chaffeensis* modulates genes related to the cell cycle, including cyclin G1 and CDC2-related protein kinases (CDKs). However, in the late stages of infection,* E. chaffeensis *upregulates cyclin E and CDC25, which activate cell replication. Thus, *E. chaffeensis *both arrests the host cell in the G1 stage during the early stages of infection to establish itself inside the cell and, in the late stages, upregulates cell proliferation to prevent the cell from dying because of progressive infection [115]. Finally, in *Ehrlichia-*infected cells, mitochondria are redistributed around *Ehrlichia morulae*, and mitochondrial metabolism is inhibited. A dormant mitochondrion may not trigger apoptosis in the infected cell. Thus, *Ehrlichia* recruits mitochondria to manipulate mitochondrial metabolism, which may result in host cell's apoptosis inhibition. Close apposition of mitochondria and *Ehrlichia* may be required for bacteria to effectively deliver proteins to mitochondria and inhibit the mitochondrial apoptosis pathway. In detail, *E. chaffeensis *downregulates JNK2 during the early stage of infection and upregulates DUSP8 and DUSP14, which dephosphorylate and inactivate JNK2. JNKs are basal in apoptosis regulation. As a matter of fact, absence of JNKs causes a defect in the mitochondrial death signalling pathway and cell apoptosis by inhibiting cytochrome *c *release [116] (Figure 12).

#### 4.2.3. Exit Mechanisms

The mechanism by which *Ehrlichia* is released from host cells or transported between cells is not fully established. Recently, it was demonstrated that *Ehrlichia* is transported to neighboring cells through the host cell filopodium during the initial stages of infection but is released by local host cell membrane rupture adjacent to the morula during later stages of infection. Filopodia are thin cell surface protrusions containing bundles of parallel actin filaments. They are designed to explore the extracellular matrix and surfaces of other cells, identifying adhesion targets or navigating to its appropriate target. So cellular exit of pathogenic microorganisms could be supported by host cells' filopodia, and it has been observed that *Ehrlichia* uses this exit mechanism. An advantage of *Ehrlichia* transport through the filopodia is that the pathogen evades the host's immune system travelling cell to cell [117]. 

#### 4.2.4. Ehrlichial Effectors and Molecular Host Interactions

Significant progress has been made in the identification of the host cell processes which *Ehrlichia *modulates. However, the effector proteins involved in manipulating these host cell processes have been largely undetermined. Recent studies have focused attention on two small groups of *Ehrlichia*-encoded proteins: those containing tandem and ankyrin repeats. These proteins appear to be effectors involved in novel, complex, and multidimensional molecular strategies to reprogram host cell defence mechanisms. Particularly, two proteins, TRP47 and Ank200, have been emphasized in recent studies, demonstrating interactions with host cell's DNA and proteins associated with distinct host cell processes [118].

**Figure 13:**
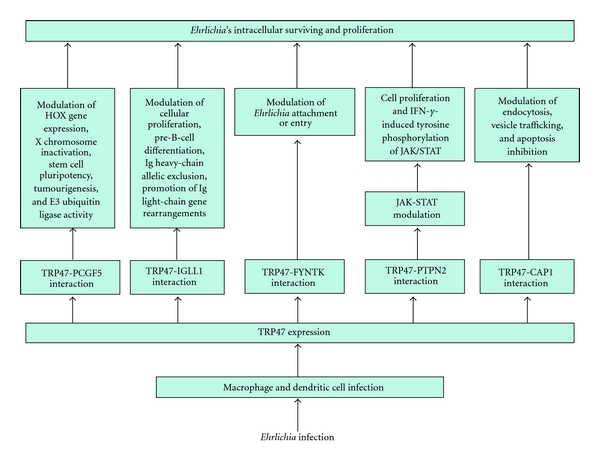
*Ehrlichia *effectors and molecular host interactions.

A recent study evaluating molecular interactions between TRP47 and host cells pointed out several interactions of this protein with several specific host cell targets. The strongest observed interaction between *Ehrlichia* and host proteins is between TRP47 and polycomb group ring finger 5 (PCGF5), which has important roles in regulation of HOX gene expression (involved in early embryonic development), X chromosome inactivation, maintenance of stem cell pluripotency, tumourigenesis, and stimulation of E3 ubiquitin ligase activity (involved in targeting proteins to be degraded within cells). Another interesting interaction is between TRP47 and immunoglobulin lambda-like polypeptide 1 (IGLL1), the surrogate light chain associated with the pre-B-cell receptor involved in signal transduction for cellular proliferation, differentiation from the pro-B-cell to pre-B-cell stage, allelic exclusion at Ig heavy-chain gene locus, and promotion of Ig light-chain gene rearrangements. Association of TRP47 with FYN tyrosine kinase, known to specifically phosphorylate caveolin-1 required for coxsackievirus internalisation and infection by caveolin-associated vesicles of epithelial cells, suggests that these proteins and their interaction might be involved in host cell attachment or entry. TRP47 also interacts with protein tyrosine phosphatase nonreceptor type 2 (PTPN2), also known as T-cell protein tyrosine phosphatase, which catalyses dephosphorylation of phosphotyrosine peptides and regulates phosphotyrosine levels in signal transduction pathways. Thus, it is involved in haematopoietic cell development and also in several human illnesses from autoimmune disease to cancer. PTPN2 has several substrates, including many JAK-STAT pathway proteins. Therefore, TRP47 might be involved not only in cellular developmental regulation but also in inhibition of IFN-*γ*-induced tyrosine phosphorylation of JAK and STAT proteins by interacting with PTPN2. Finally, TRP47 interacts with the multifunctional adenylate cyclase-associated protein 1 (CAP1), which has an active role in endocytosis, vesicle trafficking, actin turnover, and in apoptosis enhancement. Thus, in an effort to survive in the intracellular niche, *Ehrlichia* might manipulate cytoskeletal components of mononuclear phagocytes, such as actin, facilitating endocytosis and vesicle trafficking, and inhibiting apoptosis by modulating CAP1 activity [119] (Figure 13). 

Ank200, one the four ehrlichial proteins with the ankyrin (Ank) repeat motif present in eukaryotic cells and in *Ehrlichia* species, has recently been detected in host cell nuclei of *E. chaffeensis*-infected cells, where it interacts with an adenine-rich motif in promoter and intronic *Alu* elements. *Alu* elements are short, interspersed mobile DNA elements distributed in a nonrandom scheme, which comprise approximately 5–10% of the human genome, and appear to be involved in transcriptional regulation. Global analysis of Ank200 binding sites demonstrates that this protein binds to several regions distributed on nearly every chromosome by direct DNA interaction or via other DNA-binding proteins. The host cell processes targeted by Ank200 include genes associated with transcriptional regulation, structural associations with the nucleus, and apoptosis. Several Ank200 target genes have been linked to pathogenesis and immune evasion, including JAK-STAT pathway proteins (see also above) and TNF-*α*. One of primary mechanisms by which *E. chaffeensis* survives in the host cell appears to be by blocking macrophage responsiveness to IFN-*γ*. Furthermore, JAK2 transcription appears to be silenced during *E. chaffeensis* infection, at least in part, by the the ehrlichial Ank200-host DNA interaction. TNF-*α* expression is not induced early in infection, but its expression is upregulated approximately 30-fold by the day 5 after infection. *E. chaffeensis* Ank200 might contribute to TNF-*α* induction by directly binding to the promoter and upregulating gene transcription. Overproduction and high serum concentration of TNF-*α* are closely associated with fatality in severe monocytotropic ehrlichiosis [115, 120]. 

### 4.3. Immunologic Transition from Innate to Adaptive Immunity

#### 4.3.1. Ehrlichial Evasion of Innate Host Defences

*Ehrlichia* manipulates innate immune defence mechanisms, including apoptosis (see above), lysosomal fusion, production of reactive oxygen species (ROS), IFN-*γ* responsiveness by macrophages, and, in general, modulation of cell signalling pathways.

Phagolysosomes represent an important innate host defence mechanism against pathogens. *Ehrlichia* are able to inhibit fusion of phagosomes containing themselves with lysosomes and thus prevent their destruction by lysosomal enzymes. In this context, three two-component systems, named PleC-PleD, NtrY-NtrX, and CckA-CtrA, respectively, have an important role in preventing lysosomal fusion. These two-component systems, which control the response and adaptation to a variety of environmental conditions, consist of a sensor, associated with histidine kinase that detects environmental signals, and a cognate response regulator that has DNA-binding activity and regulates gene transcription. Cells treated with closantel, an inhibitor of the histidine kinase component, and incubated with *E. chaffeensis* have increased colocalisation between *E. chaffeensis* and lysosomes [121].

*Ehrlichia chaffeensis* is highly sensitive to O_2_^−^ killing and lacks genes involved in detoxification of ROS. However, it actively blocks O_2_^−^ generation and causes degradation of the NADPH oxidase subunit p22phox involved in ROS production. Degradation of p22phox is due to nonproteasomal proteolysis mediated by heat-labile factors or proteins from *E. Chaffeensis *[122].

The macrophage-activating cytokine IFN-*γ* has an important role in innate and adaptive immune responses against intracellular pathogens. Activation of macrophages by IFN-*γ* induces several antimicrobial effector mechanisms, including regulation of iron homeostasis and ROS production. As aforesaid, the ability to acquire iron is important for survival of intracellular pathogens, such as *Ehrlichia*. The antiehrlichial activity of IFN-*γ* is mediated by limiting availability of cytoplasmic iron via reduction of surface transferrin receptor. However, *E. chaffeensis* upregulates transferrin receptor expression to counteract downregulation induced by the action of IFN-*γ*. The ability of *E. chaffeensis* to block the antimicrobial mechanisms of IFN-*γ* is mediated, at least in part, by inhibition of the JAK-STAT IFN-*γ* signal transduction pathway, that is, inhibition of tyrosine phosphorylation of JAK1 and STAT1, normally induced by IFN-*γ* and mediated by the ehrlichial protein TRP47, and/or transcriptional regulation of JAK-STAT expression by the DNA-binding protein and nuclear effector Ank200 (see below) [111, 115, 123].

**Figure 14:**
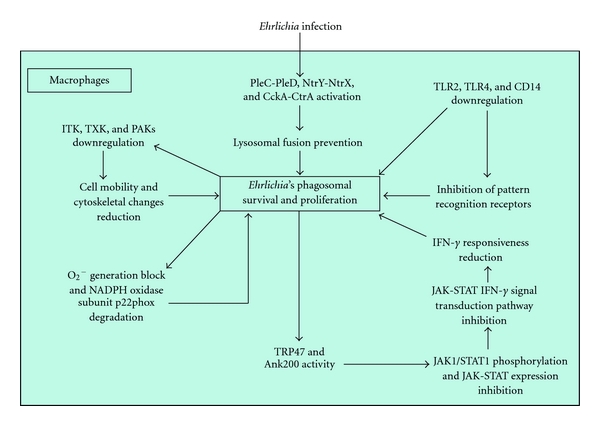
*Ehrlichia *evading innate host defences.

*Ehrlichia chaffeensis* also appears to modulate the host innate immune response by influencing other cell signalling pathways. Antimicrobial activities of macrophages become progressively less responsive during *E. chaffeensis* infection in association with downregulation of pattern recognition receptors (PRR), including TLR2, TLR4, and CD14, which act as a PRR, and the activity of the PRR transcription factor PU.1, the activation of which has been linked to the p*38* MAPK-dependent pathway. Finally, *Ehrlichia* seems to target host tyrosine kinases and phosphatases, that is, FYN and PTPN2, but the downstream processes affected by these interactions are not known. In addition, *E. chaffeensis *infection downregulates protein kinases involving cell mobility and cytoskeletal changes, such as ITK, TXK, and PAKs (i.e., PAK1, PAK2, and PAK7) (Figure 14) [113].

#### 4.3.2. Ehrlichial Immunopathological Mechanisms and Disease

During *E. chaffeensis* infection, there is a relatively low bacterial burden in blood and tissues in non-immunocompromised patients. However, the clinical manifestations, which may include fever, multiorgan failure, and adult respiratory distress syndrome, suggest that the pathogenesis of ehrlichiosis might involve immunopathological responses that are described as a toxic shock-like syndrome. Experimental lethal infection is accompanied by extremely high levels of serum TNF-*α*, high frequency of splenic TNF-*α*-producing CD8+ T cells, decreased *Ehrlichia-specific* CD4+ T-cell proliferation, low IL-12 levels in spleen, and decrease in the quantity of ehrlichial antigen-specific IFN-*γ*-producing CD4+ T_H_1 cells. Recent studies have also demonstrated that CD1d-restricted NK T cells, subsets of NK T cells that are key players in host defense against various microbial infections, might to be instrumental in induction of the immunopathological responses [124, 125].

## 5. Conclusion

Over the centuries, rickettsial, oriential, and ehrlichial diseases affected humankind all over the world, and they are still extremely widespread, remaining major health issues, with significant morbidity and mortality. They can be considered both reemerging and previously unrecognized diseases, likely owing to lack of in-depth epidemiologic surveys and application of appropriate tools for accurate diagnosis. 

Advances in our understanding of *Rickettsia*-, *Orientia*-, and *Ehrlichia*-host interactions, target cells, virulence mechanisms, structures of bacterial effector proteins, upstream signalling pathways and signal transduction systems, modulation of gene expression, host immunologic responses, apoptotic elimination of infected cells, and, finally, immunopathological basis of clinical manifestations provided new targets for therapies to block host-pathogen interactions and pathogen virulence mechanisms. 

Additional therapeutic approaches have been also identified, involving immunomodulatory therapies (i.e., antibodies-inhibiting interactions between effector proteins and host targets, inhibition of cytokine production, neutralization of cytokine effects, blocking specific cytokine receptors) and vaccine development, resulting in pathogen clearance by the immune system.

In rickettsial diseases, agents able both to quench harmful ROS production and to potentiate cellular protective mechanisms against oxygen radicals represent promising rationale for new and improved supplemental treatment. In this context, new therapeutic approaches in treating rickettsial infections and related vascular injuries are represented by the use of naturally occurring biologic antioxidants to preserve homeostasis at sites of vascular damage and by pharmacologic or genetic approaches targeting HO-1 or COX-2 in the vessel wall.
